# Vestibular dysfunction and its association with cognitive impairment and dementia

**DOI:** 10.3389/fnins.2024.1304810

**Published:** 2024-03-27

**Authors:** Cristian Aedo-Sanchez, Patricio Riquelme-Contreras, Fernando Henríquez, Enzo Aguilar-Vidal

**Affiliations:** ^1^Department of Medical Technology, Faculty of Medicine, Universidad de Chile, Santiago, Chile; ^2^Memory and Neuropsychiatric Center (CMYN), Department of Neurology, Hospital del Salvador and Faculty of Medicine, Universidad de Chile, Santiago, Chile; ^3^Laboratory of Neuropsychology and Clinical Neuroscience (LANNEC), Faculty of Medicine, Universidad de Chile, Santiago, Chile; ^4^Geroscience Center for Brain Health and Metabolism (GERO), Santiago, Chile; ^5^Laboratory for Cognitive and Evolutionary Neuroscience (LaNCE), Department of Psychiatry, Faculty of Medicine, Pontificia Universidad Católica de Chile, Santiago, Chile

**Keywords:** vestibular, dementia, Alzheimer’s disease, vestibular dysfunction, dizziness

## Abstract

The vestibular system plays an important role in maintaining balance and posture. It also contributes to vertical perception, body awareness and spatial navigation. In addition to its sensory function, the vestibular system has direct connections to key areas responsible for higher cognitive functions, such as the prefrontal cortex, insula and hippocampus. Several studies have reported that vestibular dysfunction, in particular bilateral vestibulopathy, is associated with an increased risk of cognitive impairment and the development of dementias such as Alzheimer’s disease. However, it is still controversial whether there is a causal relationship between vestibular damage and cognitive dysfunction. In this mini-review, we will explore the relationship between the vestibular system, cognitive dysfunction and dementia, hypotheses about the hypothesis and causes that may explain this phenomenon and also some potential confounders that may also lead to cognitive impairment. We will also review multimodal neuroimaging approaches that have investigated structural and functional effects on the cortico-vestibular network and finally, describe some approaches to the management of patients with vestibular damage who have shown some cognitive impairment.

## Introduction

The vestibular system plays a fundamental role in balance and spatial orientation. It is responsible for detecting movements and changes in the position of the head, both angular and linear, promotes gaze stabilization by means of the vestibulo-ocular reflex (VOR) (Delgado-García et al., 2003). Recently, it has gained attention that the study of vestibular dysfunction and its relationship with cognitive processes such as spatial orientation, memory, executive function and attention. In this sense, vestibular dysfunction has been proposed as a promoter of cognitive dysfunction and/or dementia (Bigelow and Agrawal, 2015). Cognitive functions allow us to gather, analyze and filter information from our environment that is later used by our central nervous system in our daily lives (Bigelow and Agrawal, 2015). Alzheimer’s disease (AD) is the most common cause of dementias and results in a gradual deterioration of memory, thinking, behavior and social skills. It is estimated that around 60% of AD patients suffer from a loss of spatial orientation (Fu et al., 2017). According to some studies, the entorhinal cortex is affected by the accumulation of neurofibrillary tangles of tau protein, which destroy cells in the excitatory network (Fu et al., 2017; Ying et al., 2022). In the last decade it has been proposed that vestibular dysfunction may contribute to the development of AD (Goll et al., 2012; Previc, 2013; Bigelow and Agrawal, 2015; Harun et al., 2016; Wei et al., 2020; Huang et al., 2023); however it is still less clear whether other factors concomitant with vestibular dysfunction (e.g., hearing loss or social isolation) may be contributing to cognitive impairment and also, which specific structures or pathways of the vestibular system might be involved.

## Methodology

A literature search was conducted in the PubMed database. The strategy for finding suitable articles for this review was the use of a single term. The single term used was “Cognitive dysfunction and vestibular impairment.” A total of 142 entries were obtained using this term. Articles with the following keywords were included: cognitive impairement, vestibular dysfunction, vestibular bilateral faillure, cognitive functions, hippocampus, parieto insular vestibular cortex and dementia. Both animal and human studies were included in this review. After reading the abstracts of these studies, a total of 59 articles were included for this mini review, mainly realized between 2013 and 2023.

### Vestibular pathway, hippocampus and spatial navigation

It has been reported that in humans, the hippocampus is connected to the vestibular system (via *head direction cells* and *place cells*) (Hitier et al., 2014), so its dysfunction would lead to failures in spatial navigation. When the vestibular system is damaged, the discharge rate of place cells decreases significantly. In the case of head direction cells, they have very fine tuning for the different angles of head position change, which would be lost when the vestibular system is damaged (Clark and Taube, 2012; Yoder and Taube, 2014). *Head direction cells* project vestibular information to the hippocampus, mainly to the medial entorhinal cortex and the post-subiculum (Yoder and Taube, 2014). All this information is computed by the *place cells,* generating a cognitive map for the spatial navigation (Hitier et al., 2014; Eichenbaum and Eichenbaum, 2017). In healthy subjects without vestibular impairment, goal-directed spatial navigation involves functional connections mainly between the hippocampus, retrosplenial cortex and visual cortex. This suggests the presence of a dynamic interaction between these systems to support spatial cognition (Chrastil et al., 2017). A seminal study by Brandt et al. (2005), showed that bilateral vestibulopathy, leads to hippocampal volume atrophy, using volumetric magnetic resonance imaging. The same subjects were also asked to perform a virtual version of *the Morris water maze*. In this test, these patients showed a marked deficit in spatial memory and navigation that closely matched the pattern of hippocampal atrophy (Brandt et al., 2005). In addition, patients with neurofibromatosis type 2 have been shown to have spatial memory deficits and hippocampal atrophy on MRI (Schautzer et al., 2003). These studies suggest that both the hippocampus and the vestibular system are relevant to spatial navigation. However, questions remain as to whether damage to the maculae (and graviceptic pathways) is as or more relevant than impairment of the VOR or vestibulo-spinal pathways.

### Is there a direct link between cognitive dysfunction and vestibular dysfunction?

The hypothesis that vestibular dysfunction may play a role in the pathogenesis of dementia has been gradually explored over the last decade. In 2013, the lead author and colleagues proposed the hypothesis that vestibular dysfunction may contribute to the development of AD (Previc, 2013). The main mechanism proposed is that indirect cholinergic projections from the horizontal semicircular canals to the hippocampus and its associated regions, such as the parahippocampal gyrus, posterior parietal–temporal cortex and posterior cingulate cortex (Smith, 2022). This hypothesis is supported by the fact that the central vestibular system (including the vestibular nuclei of the brainstem) contributes to the main cholinergic inputs to the hippocampus (Chapleau et al., 2016). In addition, it has been reported that bilateral vestibular dysfunction in rats also decreases cholinergic receptors in the hippocampus (Aitken et al., 2017).

In the last 10 years, it has been studied how vestibular dysfunctions might affect different cognitive domains such as attention, spatial memory, executive functions, spatial navigation or body self-awareness, and reported that vestibular dysfunction would have varying degrees of effect on cognitive abilities (Shumway-Cook and Woollacott, 2000; Uekermann and Daum, 2001; Yardley et al., 2001; Schautzer et al., 2003). Other studies have compared cognitive functions in groups with and without vestibular dysfunction. Popp et al. (2017), recruited 16 patients with unilateral vestibulopathy, 18 patients with bilateral vestibulopathy and 17 healthy controls and assessed the cognitive domains of short-term memory, executive function, processing speed and visuospatial abilities. Subjects with bilateral damage performed worse than the control group on tests of visuospatial abilities, rapid processing, memory and executive function, while subjects with unilateral damage performed worse on visuospatial abilities (Popp et al., 2017). The performance on vestibular tests has also been described *in subjects diagnosed with some degree of dementia*. Harun et al. (2016), described performance on vestibular tests in 47 subjects over the age of 55 (15 diagnosed with mild cognitive impairment and 32 with Alzheimer’s disease), using the cervical and ocular vestibular myogenic evoked potential tests (cVEMP and oVEMP, respectively) and the video-assisted head impulse test (vHIT). In this study, the absence of cVEMP was associated with a threefold increased likelihood of having Alzheimer’s disease, while a one microvolt increase in oVEMP amplitudes was associated with a decreased likelihood of not having Alzheimer’s disease. In relation to the vHIT technique, there was no significant difference in vestibulo-ocular reflex (VOR) gain between the two groups (Harun et al., 2016).

In Bigelow and Agrawal (2015) proposed a conceptual model of the mechanisms involved in cognitive dysfunction that could be generated by changes at the peripheral vestibular level. This model suggests that peripheral vestibular dysfunction (probably due to a VOR dysfunction) could lead to atrophy of brain areas associated with PICV, which could be associated with memory impairment and visuospatial abilities. In addition, postural instability and vestibulo-ocular reflex dysfunction may be associated with increased attentional demands to maintain balance, reducing the availability of cognitive resources for other tasks. It is not yet clear how vestibular dysfunctions or abnormal VOR functioning may promote the generation of cognitive dysfunctions. However, the study conducted by Harun et al. (2016) shows that damage to the saccule (assessed by cVEMP) is more likely to be related to the development of cognitive impairment than impairment of the vestibulo-ocular reflex (assessed by vHIT). This is a relevant point as impairment of the graviceptic pathways (that is, damage to the maculae) could have a greater relevance in the genesis of cognitive impairment than damage to the vestibular pathways related to the VOR. One explanation for this is that the graviceptic pathways have direct projections to the hippocampus (Smith and Zheng, 2013; Smith, 2022). Even so, these studies have not considered how certain potential confounders (social isolation, hearing loss or neurological alterations) might be influencing cognitive impairment.

### Aetiologies and confounding factors between dementias and vestibular dysfunctions: neurological alterations, social isolation and hearing loss

The above models explain and theorise the relationship between vestibular dysfunctions and the development of dementia, in a functional way. However, they do not take into account possible confounding factors related to the vestibular system, such as central nervous system tumours, hearing loss and social isolation. In the case of tumour pathologies, we can mention the neurofibromatosis type 2, wich presents vestibulocochlear nerve tumours (bilaterally), tinnitus, hearing loss, imbalance and other symptoms such as intramedullary tumours and ocular disorders (Evans, 2015). On the other hand, there are certain tumours that affect brain areas related to PIVC (such as the temporo-parietal junction) have also been reported, where subjects experience alterations in body self-perception and spatial localisation (phenomena known as *autoscopy*) (Schwabe and Blanke, 2008; Blanke, 2012; Lopez, 2013).

Another relevant potential confounding factor is social isolation and vestibular damage. It has been widely described that individuals with vestibular damage experience more feelings of anxiety, fear and even panic attacks. As a result, patients make lifestyle changes that lead to increased social isolation (Regauer et al., 2022). In general, dizziness when getting up (e.g., from a bed or chair) increases the risk of falls, which can result in injury, especially in older people (Alghwiri et al., 2013; Regauer et al., 2022). Feeling insecure when standing up, people may become reluctant to participate in social activities or leave the house, which can lead to social isolation. One way to quantify the degree of autonomy of a subject (and therefore, less social isolation) is the activities of daily living (ADLs) (Slachevsky et al., 2019; Dubbelman et al., 2020). In dementia, the assessment of ADLs is a relevant variable for its clinical diagnosis since its alterations, generated by cognitive disorders, result in the patient’s loss of autonomy and independence (McKhann et al., 2011). These ADLs may be altered when a subject with vestibular damage has a degree of social isolation, as a consequence of vestibular disorders. It has been described that patients with peripheral vestibular disorder who underwent rehabilitation improved their self-perceived ability to perform daily activities, demonstrating the relationship between ADLs and vestibular disorder (Cohen and Kimball, 2003). On the other hand, scores on scales assessing ADLs in patients with vestibular disorder have been shown to correlate with structural changes observed on MRI during compensation processes (Helmchen et al., 2011). The vestibular impairment influences the functional capacity of patients and, thus, their independence. This impairment has a real impact on a social and personal level, similar to that of patients with dementia, so there is a need to assess the ADLs of patients with vestibular disorders, which has led to the development of scales that allow doing so (Harun et al., 2016).

**Figure 1:**
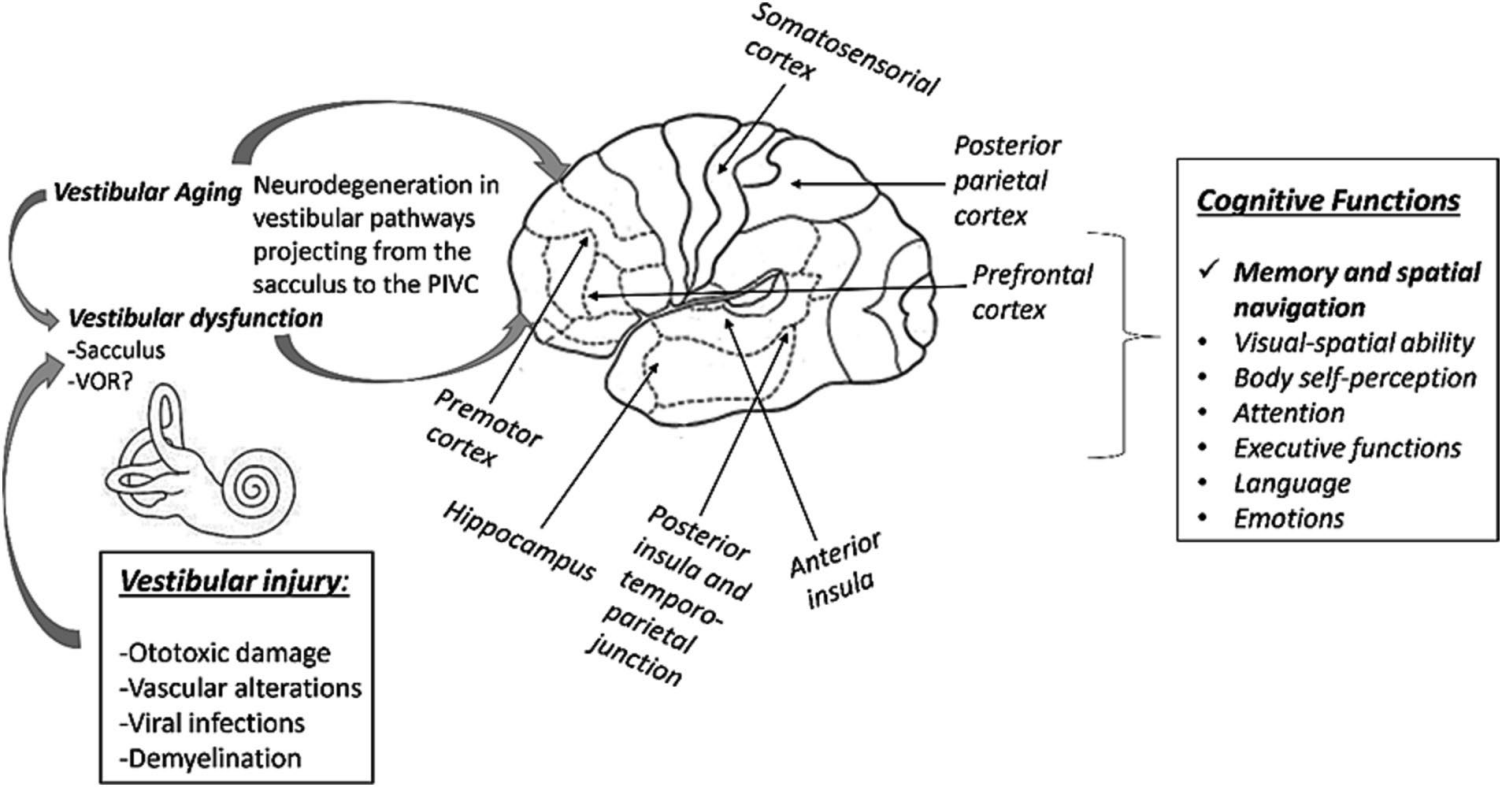
Scheme between the link between vestibular alterations and the generation of cognitive dysfunctions. A bilateral damage to the vestibular pathways (mainly from the sacculus) would project erroneous information to the hippocampus and other PIVC structures. These dysfunctions would have an impact on cognitive functions, particularly memory and spatial navigation. Damage to the VOR would apparently have little impact on the generation of cognitive deficits.

Finally, the hearing loss (especially moderate to severe) has also been described as a predictor of cognitive decline. Bilateral hearing loss is important because vestibular dysfunction, especially in older adults, is commonly associated with *age-related* hearing loss. This would suggest that hypoacusis may also contribute to cognitive deficits in these cases and that they are not exclusively due to vestibular dysfunction or aging (Smith, 2021, 2023). Some work has been done to control for both variables. Dobbels et al. (2019), showed that the performance of subjects with VPB on the *Morris water maze* test was worse than that of healthy subjects, as might be expected. However, when these subjects were controlled for degree of hearing loss, hearing loss was found to be statistically significantly associated with poorer spatial cognition. The worse the hearing of patients with BPV, the worse the spatial learning (Dobbels et al., 2019). In another study, Bosmans et al. (2022), also controlled for the effect of hearing loss to assess possible cognitive dysfunction in subjects with bilateral vestibulopathy compared to a control group. Deficits were found in immediate memory, visuospatial cognition, and attention. However, the subdomains of language and delayed memory remained normal. It was found that this cognitive loss was independent of the concomitant hearing loss of each individual with bilateral vestibulopathy (Bosmans et al., 2022). A recent study suggests that although alterations increase the risk of dementia, this would not be greater than the risk in subjects with hearing loss (Jin Lim et al., 2023). The vestibular dysfunction (like hearing loss) should be considered an independent risk factor for dementia (like hearing loss or social isolation), and that appropriate treatments, both pharmacological and appropriate vestibular rehabilitation, can reduce this risk (Figure 1).

### How can we approach to the linkage between vestibular disfunction and dementia from the neuroimaging analysis?

Electrophysiological studies in non-human primates describe a widespread vestibular cortical and subcortical network whose core area is the parieto-insular-vestibular cortex (PIVC) (Guldin and Grüsser, 1998; Brandt, 2003; Eickhoff et al., 2006; Frank and Greenlee, 2018). Neuroimaging studies (fMRI, PET and EEG) suggest that this network is bilateral, even for unilateral vestibular stimulation, It includes the posterior parietal operculum, the secondary somatosensory cortex, the inferior parietal cortex, the superior temporal cortex, the posterior insula and the premotor cortex (Ferrè and Haggard, 2020). Smith et al. (2022), leveraged structural and functional neuroimaging to characterize this extended network in healthy control participants and patients with post-concussive vestibular dysfunction (PCVD). Here, 82 regions of interest (network nodes) were identified based on previous publications, group wise differences in BOLD signal amplitude and connectivity and multivariate pattern analysis in four affective tests, one bedside cognitive screen (SAC test) and three self-report affective assessments (Beck Depression Inventory 2nd ed., Beck Anxiety Inventory and Post-traumatic Stress Disorder Checklist for the DSM-5). Group-specific “core” networks, as well as a “consensus” network comprised of connections common to all participants, were then generated based on probabilistic tractography and functional connectivity between the 82 nodes. Whereas the consensus network was comprised of affective, integrative, and vestibular nodes, PCVD participants exhibited diminished integration and centrality among vestibular and affective nodes and increased centrality of visual, supplementary motor, and frontal and cingulate eye field node (Smith et al., 2022).

Structural MRI analysis, through volumetric measurements, also can help to understand the role of multiple brain areas have in the vestibular cognition and how it can relate with dementia. Schöne et al. (2022), compared hippocampal volume, as well as supramarginal, superior temporal and postcentral gyrus in a sample of 55 patients with different conditions of peripheral vestibular dysfunction to 39 age and sex matched healthy controls. Correlations between morphometric data and visuo-spatial performance were analyzed too. Patients with different conditions of peripheral vestibular dysfunction (bilateral, chronic unilateral, acute unilateral) had no reduced total hippocampal volume compared to age- and sex-matched healthy controls. Contrary to bilateral vestibular dysfunction, chronic and acute unilateral vestibular dysfunction were associated with reduced brain volumes in the right *presubiculum* of the hippocampus and the left supramarginal gyrus. Unilateral peripheral vestibular dysfunction might lead to reduced central brain volumes that are involved in the processing of vestibular information (Schöne et al., 2022).

However, because of the distributed networks of cortical and subcortical regions involved in human vestibular processing, an anatomical identification of the hubs that conform this network is the first step to perform correct neuroimaging approaches in this field. Is in this context, Smith et al. (2023), established a compilation of existing, peer-reviewed brain atlases which collectively afford comprehensive coverage of these regions while explicitly focusing on vestibular substrates. The atlas was denoted Eagle-449 to indicate that it included 449 regions of interest related with six parcelations: anatomical cortical parcellation (Atlas A1), structural-functional cortical parcellation (Atlas A2), cerebellar anatomical parcellation (Atlas A3), thalamus parcellation (Atlas B1), brainstem and diencephalon (Atlases B2 and B3) and anatomical hypothalamus parcellation (Atlas B4) (Smith et al., 2023). Although imaging methods are the most relevant for detecting structural changes in the brain (in this particular case, due to peripheral vestibular deprivation), they also have certain limitation, which may underestimate or overestimate small hippocampal volumes. Establish a realiable method to study hippocampal structure is important in the context of vestibular dysfunction because for the evidence that implicate the effect of vestibular loss on cognitive decline, including hippocampal volume loss. As hippocampal atrophy is an important biomarker of Alzheimer’s disease, exploring vestibular dysfunction as a risk factor for dementia and its role in hippocampal atrophy is of interest (Bosmans et al., 2023).

### Can physical, clinical therapies or galvanic stimulation improve cognitive test performance in people with vestibular disorders?

Galvanic stimulation of the vestibular system (electrical stimulation of the mastoid) has been shown to improve both vestibular function itself and performance on some cognitive tests when applied (Lenggenhager et al., 2008; Dilda et al., 2012; Nguyen et al., 2021). Galvanic vestibular stimulation at the subsensory level (undetectable to the individual) has been reported to improve visuospatial, attention, memory and visuomotor functions (Dilda et al., 2012), as well as to improve individual performance on spatial working memory and mental rotation tasks. Another plausible strategy is to study the effects of clinical and/or therapeutic otoneurological treatment (vestibular rehabilitation) in this group of patients and to observe its long-term effects on cognitive performance. Zhong et al. (2023) report an improvement in cognitive performance using the Montreal Cognitive Assessment (MoCA) test in subjects with Meniere’s syndrome treated with 3, 6 and 12 months of farmacological treatment or surgery (Zhong et al., 2023). Yesantharao et al. (2023), have proposed a clinical pilot where vestibular rehabilitation therapy could improve the risk of falls. This group has proposed vestibular screening (assessment of utricular, saccular and semicircular canal function) followed by 8 weeks of therapy including horizontal gaze shifting and imaginary target exercises. They then propose to evaluate gait tests (*Berg Balance Scale and Timed Up and Go*) and cognitive tests to assess spatial cognition (Yesantharao et al., 2023).

## Conclusions, future directions and limitations

In this mini review, we have described the relationship between vestibular and cognitive dysfunction. While research describing the causal mechanisms between vestibular loss and cognitive dysfunction is still lacking, there is evidence that vestibular loss is associated with cognitive dysfunction, in particular with memory and spatial navigation. However, basic studies to better understand this phenomenon are still lacking. For example, studies at the cellular level and/or biomarkers to establish a molecular hypothesis between the possible relationship between vestibular dysfunction and cognitive impairment. In the future, it would be very interesting to propose or conduct in future longitudinal cohort studies comparing cognitive function before and after bilateral vestibulopathy in a large cohort of individuals. This will allow us to see what changes may occur at a cognitive level as the vestibular pathology changes from unilateral to bilateral. We consider that early testing in a clinical consultation would allow an earlier diagnosis of these conditions. Vestibular dysfunction (similar to hearing loss) should be considered an independent risk factor for dementia and in appropriate treatments, whether pharmacological or vestibular rehabilitation. In addition, it would be highly desirable to consider including tests of spatial navigation ability in their evaluations, both in subjects with risk factors of family or genetic history of dementia, and in subjects with bilateral vestibular dysfunction. This test could be complemented by fMRI and volumetric MRI analysis to understand the organization of vestibular networks in the brain and their alteration in vestibular dysfunction.

In conclusion, we can point to causal evidence linking vestibular dysfunction (mainly bilateral) with cognitive dysfunction, mainly hippocampal damage and alterations in spatial navigation.

### Limitations

As limitations in this mini review, we have not been able to elaborate more extensively on other potential confounders for the development of dementia, such as the effect of cardiovascular disorder on vestibular function and it’s relationship to cognitive decline, consumption of alcohol on vestibular function and exercise and physical activity, vestibular disorders it’s relationship to cognitive decline.

## Author contributions

CA-S: Conceptualization, Investigation, Writing – original draft. PR-C: Conceptualization, Investigation, Writing – review & editing. FH: Investigation, Writing – review & editing. EA-V: Conceptualization, Investigation, Supervision, Writing – review & editing.
